# Sophorolipid Biosurfactant Can Control Cutaneous Dermatophytosis Caused by *Trichophyton mentagrophytes*

**DOI:** 10.3389/fmicb.2020.00329

**Published:** 2020-03-12

**Authors:** Suparna Sen, Siddhartha Narayan Borah, Raghuram Kandimalla, Arijit Bora, Suresh Deka

**Affiliations:** ^1^Environmental Biotechnology Laboratory, Resource Management and Environment Section, Life Sciences Division, Institute of Advanced Study in Science and Technology, Guwahati, India; ^2^Drug Discovery Laboratory, Life Sciences Division, Institute of Advanced Study in Science and Technology, Guwahati, India; ^3^Department of Bioengineering and Technology, Institute of Science and Technology, Gauhati University, Guwahati, India

**Keywords:** biosurfactant, sophorolipid, *Trichophyton mentagrophytes*, dermatophytosis, antibiofilm activity, ultramicroscopy, topical application

## Abstract

*Trichophyton mentagrophytes*, a zoophilic species, is one of the most frequently isolated dermatophytes in many parts of the world. This study investigated the efficacy of a sophorolipid (SL-YS3) produced by *Rhodotorula babjevae* YS3 against dermatophytosis caused by *T. mentagrophytes*. SL-YS3 was characterized by gas chromatography–mass spectrometry (GC–MS) and ultra-performance liquid chromatography, coupled with electrospray mass spectrometry (UPLC-ESI-MS). SL-YS3 comprised of six different fatty acids as the hydrophobic components of constituent congeners and sophorose as the hydrophilic component. Inhibitory effects of purified SL-YS3 against hyphal growth was found to be 85% at a 2 mg ml^–1^ concentration, and MIC was 1 mg ml^–1^. Microscopic examination with scanning electron microscopy (SEM), atomic force microscopy, and confocal laser scanning microscopy (CLSM) revealed that SL-YS3 exerts its effect by disrupting cell membrane integrity causing cell death. SL-YS3 was also effective in reducing the biofilms formed by *T. mentagrophytes*, which was observed spectrophotometrically with crystal-violet staining and further validated with SEM and CLSM studies of treated biofilms. *In vivo* studies in a mouse model of cutaneous dermatophytosis involving macroscopic observations, percent culture recovery from skin samples, and histopathological studies showed that SL-YS3 could effectively cure the infected mice after 21 days of topical treatment. Terbinafine (TRB) was used as a standard drug in the experiments. We demonstrate, for the first time, the antidermatophytic activity of a sophorolipid biosurfactant. The findings are suggestive that SL-YS3 can be formulated as a novel antifungal compound to treat cutaneous mycoses caused by *T. mentagrophytes*.

## Introduction

*Trichophyton mentagrophytes* is a zoonotic dermatophyte found in a variety of environments and is especially dominant in clinical cases of tinea pedis and onychomycosis, with a prevalence of 1.5–55.4% (Ameen, 2010; Agarwal et al., 2014). *T. mentagrophytes* is a common cause of dermatophytosis in companion animals, livestock, and wildlife, and is therefore considered a public health hazard because of the high possibility of zoonosis (Drouot et al., 2009). In the last decade, there has been an epidemiological renaissance of zoophilic dermatophytes. The existing treatments for these infections are reliant on a few synthetic antifungal agents (particularly azoles and allylamines). The clinical values of these agents have been limited due to their narrow spectrum of action, toxicity, and the recurrence of infection (Soberón et al., 2007). The selective pressure exerted by the constant use of the conventional antifungal agents, or at times partial eradication due to incomplete treatment courses or other factors, might eventually lead to the emergence of a predominant resistant strain in the population (Dogra and Uprety, 2016). Therapeutic failures with oral and topical formulations of azoles and allylamines, especially with terbinafine, has been recorded in patients with tinea pedis or onychomycosis, mainly due to the emergence of resistant isolates (Mukherjee et al., 2003; Yamada et al., 2017). Several studies have outlined the molecular basis of such resistance, for instance, Yamada et al. (2017) reported terbinafine resistant strains (∼1%) collected from patients with tinea pedis or unguium, all of which were found to harbor missense mutations in the SQLE gene, leading to single amino acid substitutions at one of four positions (Leu393, Phe397, Phe415, and His440) of the SQLE enzyme. Singh et al. (2018) reported 32% terbinafine resistant *T. interdigitale* isolates obtained from cases of tinea corporis or tinea cruris, exhibiting single point mutations (Leu393Phe or Phe397Leu) in the SQLE gene resulting in high MICs. Similarly, Rudramurthy et al. (2018) reported a T1189C transition in the SQLE gene leading to a change of phenylalanine at the 397th position to leucine (Phe397Leu) in *T. interdigitale* and *T. rubrum* isolates. In addition, Santos et al. (2018) observed that terbinafine resistance of *T. rubrum* was linked to the salA gene, encoding salicylate 1-monooxygenase, where increased expression of salA resulted in resistance. Thus, there is a need for the search of new sources of anti-dermatophytic compounds, which are efficacious, cheaper, and safer/non-toxic alternatives to their synthetic counterparts. Despite the high incidence of dermatophyte infections, few investigations have been performed to better understand these infections, and most of the studies were based on *in vitro* experiments (De Pauw, 2000).

Biological surface-active agents or biosurfactants are produced by bacteria, yeasts, and fungi either extracellularly or bound to their cell walls (Rodrigues et al., 2006). Sophorolipids (SLs) are glycolipid types of biosurfactants synthesized mainly by yeasts related to the *Starmerella*, and *Rhodotorula* clades. SLs are produced as a mixture of both acidic and lactonic forms of the molecule. They are gaining attention as promising natural surfactants because of several advantages over chemical surfactants, such as low toxicity, inherently good biodegradability, and ecological acceptability (Daverey and Pakshirajan, 2009). SLs find applications in the cleaning, environmental, and food industry as well as in personal care, cosmetic, and pharmaceutical sectors (Baviere et al., 1994; Hall et al., 1996; Mulligan et al., 2001). They exhibit low cytotoxicity, and their use in food and pharmaceutical industries has been approved by the U.S. Food and Drug Administration (FDA) (Joshi-Navare and Prabhune, 2013). Additionally, SLs reportedly cause very low acute toxicity (safe up to 5.8 to 6.6 g/kg on subcutaneous administration in mice), and skin irritation, skin sensitization when studied in rats, mice, rabbits, and guinea pigs (Ikeda et al., 1986). SLs have been reported to stimulate the dermal fibroblast metabolism and collagen neosynthesis, inhibit free radical and elastase activity, possess macrophage-activating and fibrinolytic properties and act as desquamating agents making them attractive candidates for dermatological applications (Van Bogaert et al., 2007). Their compatibility with skin tissue and their tissue healing properties make them attractive candidates for dermal applications (Lydon et al., 2017). They have also been explored for antimicrobial properties against bacteria, phytopathogenic fungi, and reportedly have anti-HIV and sperm-immobilizing activities (Gi, 2004; Shah et al., 2005b; Yoo et al., 2005; Sen et al., 2017). However, SLs are relatively unexplored for their role in controlling filamentous fungi causing diseases in humans or animals.

The current study was, therefore, performed to evaluate the *in vitro* and *in vivo* antimycotic activity of an SL biosurfactant extracted from the *Rhodotorula babjevae* strain YS3, against *T. mentagrophytes*, as a non-toxic alternative to conventional synthetic antifungals. To the best of our knowledge, this is the first report documenting the antifungal activity of SLs against dermatophytes.

## Materials and Methods

### Microorganisms

In the present study, the yeast strain *R. babjevae* YS3 was used for biosurfactant production, which was reported to produce SL for the first time in our earlier study (Sen et al., 2017). The strain was maintained in 20% (v/v) glycerol stocks stored at −80°C. A fungal culture of *T. mentagrophytes* (NCCPF 800049) was obtained from the National Culture Collection of Pathogenic Fungi (NCCPF), India. The fungus was cultured in Sabouraud Dextrose broth/agar (SDB/SDA; HiMedia, India), and the microconidial suspension was stored at −80°C in 30% glycerol (v/v) for maintenance. The working cultures were maintained in SDB/SDA plates at 4°C and subcultured every 2 weeks.

### Characterization of SL-YS3

*Rhodotorula babjevae* YS3 was cultured in Bushnell-Haas medium (BHM; Himedia, India) at 19 ± 2°C with 10% (w/v) glucose as the carbon source for the production of SL (SL-YS3) used in this study (Sen et al., 2017). A detailed description of the culture conditions and partial characterization of column purified SL-YS3 by attenuated total reflectance–Fourier transformed infra-red spectroscopy (ATR-FTIR), and liquid chromatography-electrospray ionization-mass spectrometry (LC-ESI-MS) analyses have been reported in our previous study (Sen et al., 2017). Additional data from GC–MS analyses of the fatty acid moieties, along with ultra-performance liquid chromatography-electrospray ionization-mass spectrometry (UPLC-ESI-MS) analyses of the sugar component, are provided in this study. For GC–MS analysis, the fatty acid component of SL-YS3 was first converted to fatty acid methyl esters (FAME) and analyzed using GC/MS (TQ8030, Shimadzu, Japan) with an EB-5MS capillary column (30 m × 0.25 mm × 0.25 μm) (Smyth et al., 2010). Briefly, methanol containing 1% sulfuric acid (2 ml) was added to approximately 50 mg of SL-YS3 and heated at 100°C for 40 min. The reaction product was then extracted with 5 ml cyclohexane with 5 ml 50 gl^–1^ NaCl. The cyclohexane layer was then dried under a stream of nitrogen. The injection temperature was set at 275°C, and a column oven temperature was set at 65°C for 5 min ramping at 10°C min^–1^ to 290°C (held for 5 min). Helium was used as the carrier gas at a flow rate of 1 ml min^–1^. MS scan was performed in the range of m/z 10-350 and analyzed with GC–MS solution software (version 4). Compounds were identified using the NIST 11 library database.

The monosaccharide composition of the sugar moiety in SL-YS3 was analyzed by UPLC-ESI-MS (UHPLC system coupled to a triple quadrupole Orbitrap MS/MS, Thermo Scientific, Waltham, MA, United States) (White et al., 1990). SL-YS3 (10 mg) was subjected to alkaline hydrolysis to free the intact sugar moiety from esterified acyl groups followed by acid digestion (1 ml of 2M sulfuric acid) at 90°C for 6 h to yield monosaccharides only. The aqueous phase containing sugars was dried under a stream of nitrogen and then reconstituted with 1 ml of acetonitrile/water (80:20 v/v). Mass spectral analysis was performed using electrospray ionization (ESI) in the positive mode to detect individual monosaccharides. A 2 μl sample aliquot was injected into a reversed-phase Hypersil Gold C_18_ selectivity column (2.1 mm × 150 mm, particle size 1.9 μm) using a gradient of water + 0.1% formic acid (solvent A) and acetonitrile (solvent B) as the mobile phase at 40°C at a flow rate of 0.2 ml min^–1^. MS scans were performed in the scan range of m/z 66–1000 and analyzed using Thermo Scientific Xcalibur software (version 2.3.0.1765). During both GC–MS and UPLC-ESI-MS analyses, a commercially available SL, 1,4′′-sophorolactone 6′,6′′-diacetate (SL-S, Sigma-Aldrich, United States) was used as the reference standard.

### Antifungal Susceptibility Testing

Antifungal susceptibility of *T. mentagrophytes* against SL-YS3 was tested using the broth microdilution method as per the Clinical and Laboratory Standards Institute (CLSI) M38-A2 reference for filamentous fungi (Clinical and Laboratory Standards Institute (CLSI), 2002). The conidial suspension was prepared in RPMI-1640 (0.5 × 10^4^ to 5 × 10^4^ CFU ml^–1^). SL-S and terbinafine (TRB; Sigma-Aldrich, United States) were used as reference standards. Double strength stock solutions of the test compounds were prepared, and serial twofold dilutions were made in RPMI-1640 medium buffered to pH 7.0 with 0.165M 3–(*N*–morpholino) propanesulfonic acid (MOPS) buffer (both from Sigma-Aldrich St. Louis, MO, United States). A 100 μl of the prepared conidial suspension was added to each well of 96-well microtiter plates containing 100 μl of test compounds. Growth and sterility controls were also included, and the plates were incubated at 25°C for 96 h. The absorbance of each well was measured at 600 nm using a Multimode Reader (Thermo Scientific). The concentration showing no growth was considered as the minimum inhibitory concentration (MIC).

For evaluation of mycelial inhibition, a 6 mm plug of *T. mentagrophytes* in SDA was transferred to fresh plates containing SL-YS3 (0.5 to 4 mg ml^–1^) and incubated at 25°C (Borah et al., 2015) for 7 days. Control plates were prepared with SDA without a test compound. Growth inhibition was expressed as:

$$\mathrm{Inhibition}\left(\%\right)=\frac{\left(D_{C}-D_{t}\right)}{D_{C}}\times 100$$

Where, D_c_ = radial diameter of colonies on control plates, D_t_ = radial diameter of colonies on SL-YS3 amended plates.

### Microscopic Evaluation of the Anti-mycelial Effect of SL-YS3

Mycelia of *T. mentagrophytes* were excised from active cultures, treated with SL-YS3 (1 mg ml^–1^) and TRB (1 mg ml^–1^; positive control) for 7 days at 25°C, and then subjected to microscopic observations. Mycelia without any treatment served as a negative control.

For scanning electron microscopy (SEM), mycelia were excised and fixed in 2.5% glutaraldehyde (v/v) in phosphate-buffered saline (PBS) overnight at 4°C. After washing the samples thoroughly in PBS to remove the excess fixative, they were dehydrated in graded ethanol series (30–100% v/v) for 15 min each. The samples were then mounted on stubs over carbon tape and sputter-coated with gold-palladium and observed in a field-emission scanning electron microscope (FE-SEM; Zeiss, Σigma, Germany) (Borah et al., 2016).

Mycelial topography was studied in semi-contact mode using an atomic force microscope (AFM; NTEGRA prima, NT-MDT Spectrum Instruments, Moscow, Russia), equipped with a silicon cantilever (spring constant 0.03 N/m) and a scanner (100 μm × 100 μm × 5 μm scan range). Images were analyzed with Nova software version 1.1.0.1780.

For confocal laser scanning microscopy (CLSM), the treated mycelia were excised and then stained with 0.1 mg ml^–1^ propidium iodide (PI; Sigma-Aldrich) for 15 min, washed in PBS, and visualized in a confocal microscope (Leica TCS SP8 with VIS Laser Leica Microsystems, Germany) (Sen et al., 2019).

### Biofilm Susceptibility Testing

#### Disruption of Pre-formed Biofilm

*Trichophyton mentagrophytes* biofilms were formed in 96-well flat-bottom microtiter plates, according to Costa-Orlandi et al. (2014). Briefly, microconidial suspension (1 × 10^6^ CFU ml^–1^) was prepared in RPMI-1640 medium and inoculated in appropriate wells except in those for sterility control. After incubation at 28°C for 96 h, the medium was decanted, and the wells washed thoroughly with sterile PBS to remove any remaining planktonic cells. Serially double diluted solutions of SL-YS3 and TRB (standard drug) prepared in RPMI-1640 were added to the biofilms and incubated for 24 h. Control wells contained only 100 μl RPMI-1640 without SL-YS3 or TRB. After incubation, the wells were washed with PBS, and 100 μl of 0.5% crystal violet (CV) solution was added at room temperature to stain the biofilms for 15 min. The wells were washed with sterile water to remove excess stain, and biofilms were decolorized by adding 100 μl of 95% ethanol solution in each well. The ethanol solution was gently homogenized with a pipette until the rest of the CV was completely dissolved (∼1 min). Finally, the solution from each well was transferred to a new 96-well plate and then read in a Multimode Reader at 570 nm. The antibiofilm activity was calculated as:

Biofilmformation(%)=Treated⁢OD⁢ 570⁢nmControl⁢OD⁢ 570⁢nm×100

#### SEM and CLSM

Scanning electron microscopy and CLSM were performed to visualize the effect of SL-YS3 on pre-formed biofilms of *T. mentagrophytes*, as described previously (Costa-Orlandi et al., 2014; Borah et al., 2019). Biofilms were formed on pre-sterilized glass coverslips placed in 24-well plates containing RPMI-1640 medium inoculated with 1 × 10^6^CFU ml^–1^ cell suspension for 96 h. The coverslips were washed with PBS and treated with 2 × MIC concentrations of SL-YS3 and TRB. Untreated samples served as control.

For SEM, the coverslips were washed with PBS thrice and fixed in 2.5% (v/v) glutaraldehyde in PBS (0.1M, pH 7.5) at 4°C. After the PBS washes, to remove any traces of fixative, the samples were dehydrated in an increasing gradient of ethanol (30–100% v/v), mounted on stubs over carbon tapes, metalized with gold-palladium, and observed in a field emission scanning microscope (FE-SEM, Zeiss, Σigma, Germany).

For CLSM, fungal biofilms post-treatment were gently washed with PBS, stained with 100 μg ml^–1^ propidium iodide (PI; Sigma-Aldrich, United States), for 15 min and viewed in a confocal microscope (Leica TCS SP8 with VIS Laser Leica microsystems, Germany).

### *In vivo* Study

#### Animal Housing and Care

Equal numbers of male and female Swiss albino mice (25–30 g weight, approximately 8 weeks old) were randomly selected and maintained in the animal house facility at IASST under a controlled temperature (22 ± 2°C), with relative humidity (60–70%), and a 12:12 h light–dark cycle. The experimental protocol was approved (IASST/IAEC/2016-17/05) by the Institutional Animal Ethics Committee (IAEC) of IASST, Guwahati before starting the experiments and was performed using the guidelines of the Committee for the Purpose of Control and Supervision of Experiments on Animals (CPCSEA), Government of India, on animal experimentation. Mice were individually housed in polypropylene cages and fed a rodent pellet diet (Provimi Animal Nutrition Pvt. Ltd., India) and water *ad libitum*.

#### Dermal Infection and Treatment

For induction of trichophytosis, animals were anesthetized with a cocktail of ketamine (80 mg kg^–1^) and xylazine (10 mg kg^–1^) (Ghannoum and Rice, 1999). Dorsal portions of the animals were shaved and abraded at a diameter of 1.5 cm and infected with 0.2 ml conidial suspension of *T. mentagrophytes* at 1 × 10^7^ CFU ml^–1^. The animals were divided into four groups, each comprising six animals: Group I – uninfected, untreated control; Group II – infected, untreated control; Group III – topical treatment with 1 mg ml^–1^ (w/v) TRB; Group IV – topical treatment with 1 mg ml^–1^ (w/v) SL-YS3. After 72 h (after the establishment of infection), treatment was initiated once a day and was continued for 21 days.

#### Fungal Culture Recovery

Skin scrapings were collected from each infected site at intervals of 7 days, cultured in SDA plates, and incubated at 25°C for 7 to 8 days. The skin scrapings that yielded fungal growth were considered to be culture positive, and animals with one or more culture-positive skin scraping was considered to be fungus positive (Kalita et al., 2015).

Culture recovery was calculated as:

$$\mathrm{Culture}\mathrm{recovery}\left(\%\right)=\frac{\mathrm{Total}\,\mathrm{no}.\mathrm{of}\,\mathrm{culture}\,\mathrm{positive}\,\mathrm{sites}\,\mathrm{in}\,\mathrm{each}\,\mathrm{set}}{\mathrm{Total}\,\mathrm{no}.\mathrm{of}\,\mathrm{sites}\,\mathrm{in}\,\mathrm{each}\,\mathrm{set}}\times 100$$

#### Histopathology

For evaluation of the histopathological changes, the animals were euthanized by cervical dislocation at the end of the 21-day treatment period, and skin biopsy samples were fixed in neutral buffered formalin. Paraffin-embedded tissues were sectioned (4–5 mm) and stained with hematoxylin and eosin (H&E) and Periodic acid-Schiff (PAS) stain for light microscopy. The pathological changes were examined under a light microscope (Clinical and Laboratory Standards Institute (CLSI), 2002).

### Statistical Analyses

All the experiments were conducted with at least three replicates and repeated once. The results are expressed as mean ± SD. The *in vitro* antifungal effect of SL-YS3 and culture recovery (%) from the treatment groups of the *in vivo* experiments were statistically validated using a one-way analysis of variance (ANOVA) followed by the pairwise least significant difference (LSD). Differences were considered significant at *P* < 0.05. Statistical analyses were performed using SPSS Statistics v22.0 (IBM, Corp., United States).

## Results

### Compositional Analyses of SL-YS3

**FIGURE 1:**
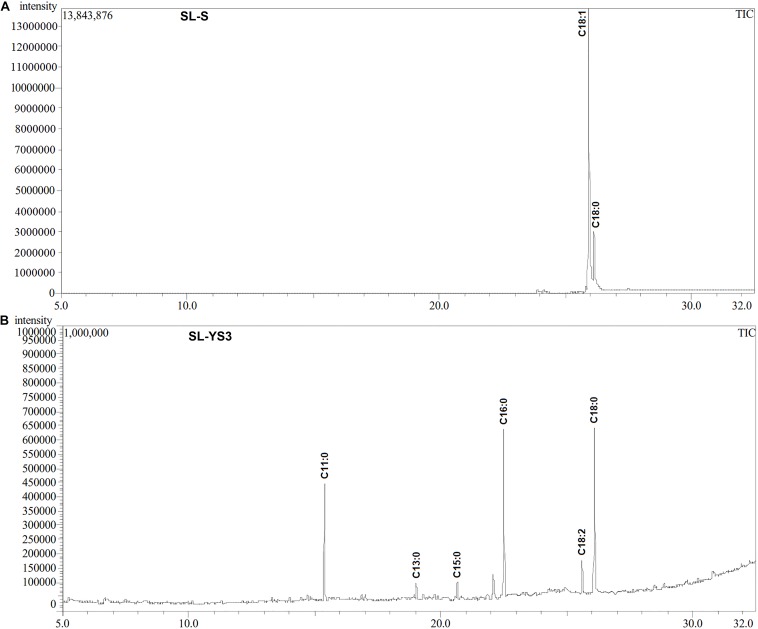
Gas chromatography profiles (TIC) of the fatty acid methyl esters (FAMEs) of the **(A)** standard sophorolipid (SL-S) and **(B)** the sophorolipid produced by *Rhodotorula babjevae* YS3 (SL-YS3).

**FIGURE 2:**
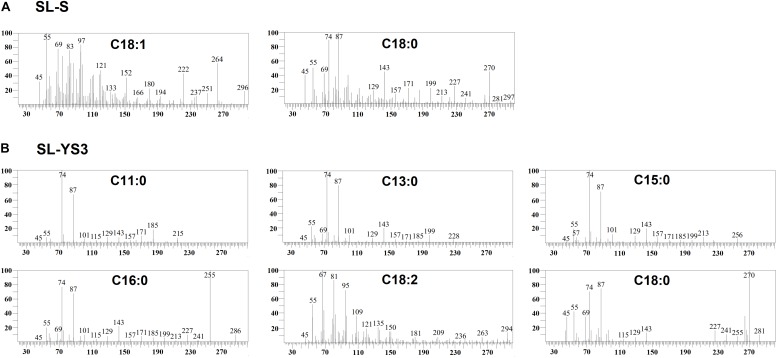
Mass spectra of the fatty acids present in the constituent congeners derivatized to fatty acid methyl esters (FAMEs) **(A)** commercially available sophorolipid 1′,4′′-Sophorolactone 6′,6′′-diacetate (SL-S) **(B)** sophorolipid produced by *R. babjevae* YS3 (SL-YS3) as identified by GC–MS analysis.

Table 1 enlists the lactonic and acidic SL homologs present in SL-YS3 and SL-S, as revealed by LC-ESI-MS analyses reported in our previous study (Sen et al., 2017). Accordingly, SL-YS3 comprises five lactonic and two acidic homologs, whereas SL-S contains only two lactonic homologs. The chromatograms obtained from the GC–MS analyses of the lipidic side chains (hydrophobic moieties) of SL-YS3 and SL-S are presented in Figure 1. Mass spectral analysis by comparison with the authentic standard and NIST library revealed that SL-YS3 was composed of congeners containing six different fatty acids: 11-hydroxyundecanoic acid (C11:0), tridecanoic acid (C13:0), pentadecanoic acid (C15:0), 16-hydroxyhexadecanoic acid (C16:0), 9,12-octadecadienoic acid (Z,Z)- (C18:2), and 17-hydroxyoctadecanoic acid (C18:0). In comparison, SL-S was found to contain two fatty acids: 9-octadecenoic acid (C18:1) and 17-hydroxyoctadecanoic acid (C18:0). The GC–MS spectra of the fatty acids present in SL-YS3 and SL-S are presented in Figure 2.

**TABLE 1:** The list of lactonic and acidic sophorolipid (SL) homologs present in the test SL produced by *Rhodotorula babjevae* YS3 (SL-YS3) and the reference standard 1′,4′′-Sophorolactone 6′,6′′-diacetate (SL-S, Sigma-Aldrich, Burlington, MA, United States) along with their relative abundance (%) as detected by LC-ESI-MS in positive mode.

**SL sample^∗^**	**SL type**	**Homolog**	**Molecular**		**Relative abundance (%)**
			**Formula**	**Mass**	

**SL-YS3**	Lactonic	LS-C_13__:__1_	C_25_H_42_O_12_	534.59	7.29
		LS-C_15__:__3_	C_27_H_42_O_12_	558.61	21.07
		LS-C_16_	C_28_H_50_O_12_	578.69	30.22
		LS-C_18__:__2_	C_30_H_50_O_12_	602.71	14.64
		Ac_2_LS-C_18_	C_34_H_58_O_14_	690.81	1.36
	Acidic	AS-C_11_	C_23_H_42_O_13_	526.57	3.15
		AS-C_13__:__1_	C_25_H_44_O_13_	552.61	12.78
**SL-S**	Lactonic	Ac_2_LS-C_18__:__1_	C_34_H_56_O_14_	688.80	72.31
		Ac_2_LS-C_18_	C_34_H_58_O_14_	690.81	22.47
	Acidic	Nil	NA	NA	NA

*LS, lactonic SL; AS, acidic SL; Ac, acetyl group. ^∗^The ESI mass spectra of the samples related to the information provided in this table have been previously published (Sen et al., 2017).*

**FIGURE 3:**
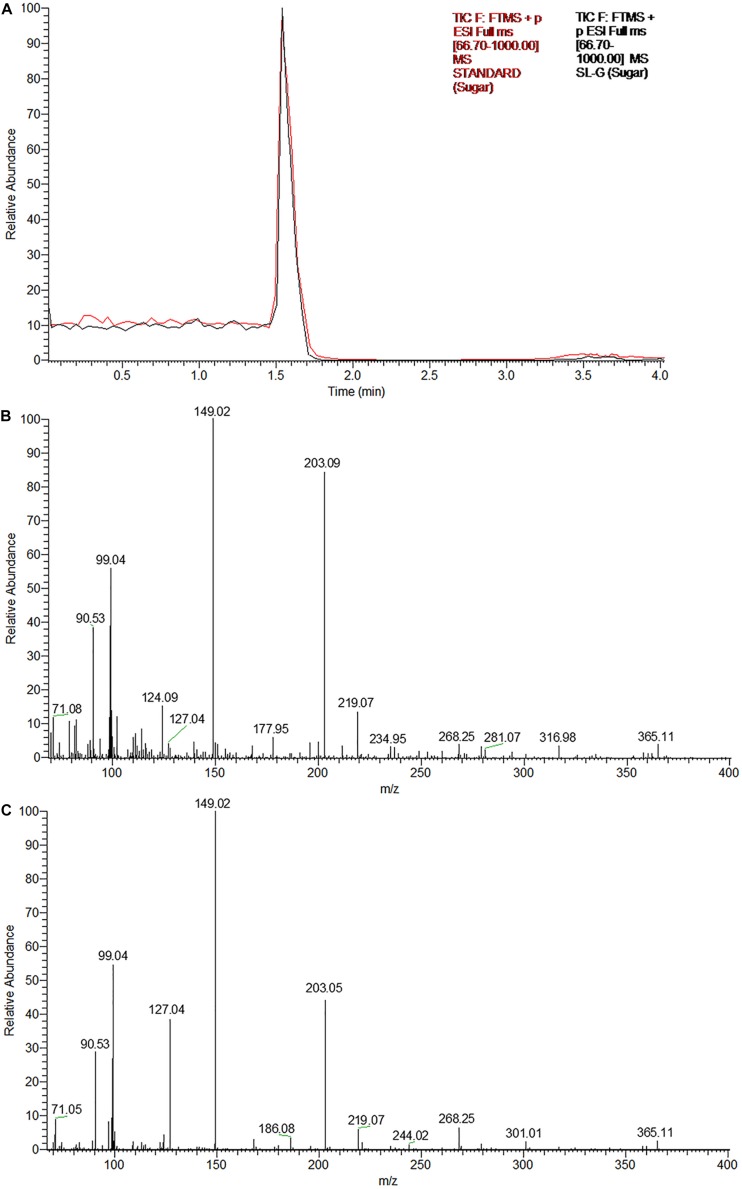
The glycan part of the biosurfactant produced by *R. babjevae* YS3 (SL-YS3) is sophorose. **(A)** UPLC chromatogram (TIC) of the sugar component of the sophorolipid under study (SL-YS3, black) in comparison with that of the standard sophorolipid (SL-S, red). The single peak with the same retention time (Rt) indicates the same sugar composition of SL-YS3 and SL-S. The positive ESI-MS spectra of **(B)** SL-S and **(C)** SL-YS3 exhibited sodiated adducts of glucose monomer [M + Na] + and dimer [2M + Na-H_2_O] + at m/z 203 and m/z 365 respectively.

The UPLC chromatograms of the sugar component (hydrophilic moiety) of both the samples yielded a single peak having the same retention time (Figure 3A). Subsequent ESI-MS analyses confirmed, the same as glucose, the constituent monosaccharide of sophorose in an SL sample (Figures 3B,C). The peak at m/z 203 corresponded to the monosodiated adduct ion, [M + Na]^+^ of glucose. The base peak at m/z 149 could be attributed to the fragment ion generated after the loss of a hydroxymethyl group (M-CH_2_OH), while the small peak at m/z 365 represented the monosodiated glucose dimer adduct after the loss of a water molecule [2M + Na-H_2_O]^+^.

### *In vitro* Susceptibility Screening

**FIGURE 4:**
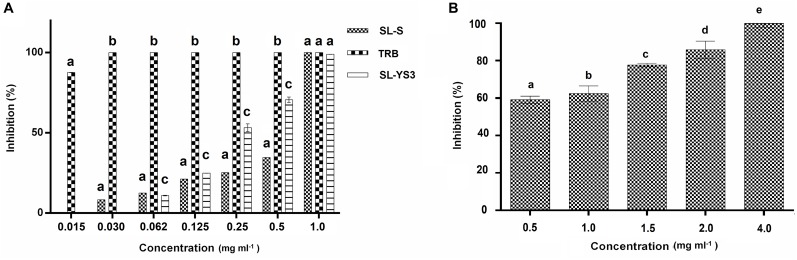
Antifungal activity of sophorolipid produced by *R. babjevae* YS3 (SL-YS3) against *Trichophyton mentagrophytes in vitro*. **(A)** Dose-dependent effect of purified SL-YS3, sophorolipid standard 1, 4′′-sophorolactone 6′,6′′-diacetate (SL-S), terbinafine (TRB) against spores of *T. mentgrophytes* as determined according to CLSI guidelines (M38 A-2). **(B)** Inhibition of mycelial growth of *T. mentagrophytes* by purified SL-YS3. Data are percentage of the mean of triplicates with respect to control. Error bars represent standard deviation. Different letters within each concentration indicate significantly different values as per ANOVA-LSD.

Broth microdilution assay was performed for the initial characterization of the antifungal activity. SL-YS3, SL-S, and TRB exhibited complete inhibition of spore germination (MIC) at concentrations 1.0, 1.0, and 0.031 mg ml^–1^, respectively (Figure 4A). Figure 4B depicts the concentration-dependent inhibition of mycelial growth. It was observed that a higher concentration of SL-YS3 was required to suppress the mycelial growth as compared to spore germination in a concentration-dependent manner. An inhibition of 62% was noted against the mycelia at the MIC (1 mg ml^–1^). At the highest concentration of SL-YS3 tested, i.e., 4 mg ml^–1^, complete inhibition of mycelial growth was recorded.

### Microscopic Evaluation of the Antimycelial Effect of SL-YS3

**FIGURE 5:**
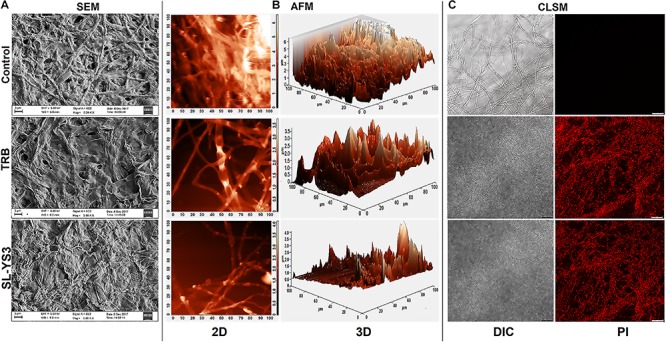
Microscopic observation of the effect of the sophorolipid produced by *R. babjevae* YS3 (SL-YS3) on mycelial integrity of *T. mentagrophytes*. **(A)** Scanning electron micrographs of the mycelial damage induced by SL-YS3 and terbinafine (TRB) treatment. Magnification = 5000, scale bar = 2 μm. **(B)** Atomic force microscopic images to visualize the topographical changes induced by SL-YS3 and TRB treatment (1 mg ml^–1^) in comparison to the untreated control mycelia. Images were acquired after 24 h of treatment. Total scanning area for the images are 100 μm × 100 μm. **(C)** Confocal microscopic images to determine the uptake of propidium iodide (PI) in the mycelia treated with SL-YS3 and TRB at a concentration of 1 mg ml^–1^ (w/v) as compared to the untreated mycelia. Scale bar = 25 μm.

Figure 5A illustrates the morphological changes as observed in SEM in the mycelia of *T. mentagrophytes* post-treatment with SL-YS3 and TRB. Severely damaged mycelia with irregular morphology, flattened hyphae along with ruptured surfaces were observed. On the other hand, untreated control hyphae had a uniform cell morphology and no visible damage.

Atomic force microscopy was carried out to understand the topographical alterations of the fungal cells being treated with SL-YS3. Figure 5B represents the three-dimensional micrographs of the treated mycelia, which shows an uneven biomass and reduced cell height. The reduced cell height (as seen from the Z-axis in the 3-D images) is due to the flattening of hyphal structures resulting from the exudation of cellular contents after cell lysis and loss of architectural support. In contrast, the untreated mycelia depicted uniform cell height and distribution. Patches of roughness were observed on the treated samples due to incomplete exudation of the cytoplasmic fluid. The surface of the control hyphae was also observed to be smoother compared to the SL-YS3 treated ones.

Confocal laser scanning microscopy was performed to determine the cell membrane damage as measured by the uptake of PI. In the control samples, no fluorescence could be detected. However, after exposure to SL-YS3 and TRB, increased fluorescence was observed, indicating the uptake of PI due to loss of cell membrane integrity (Figure 5C).

### Biofilm Susceptibility Testing

#### Disruption of Pre-formed Biofilm

**FIGURE 6:**
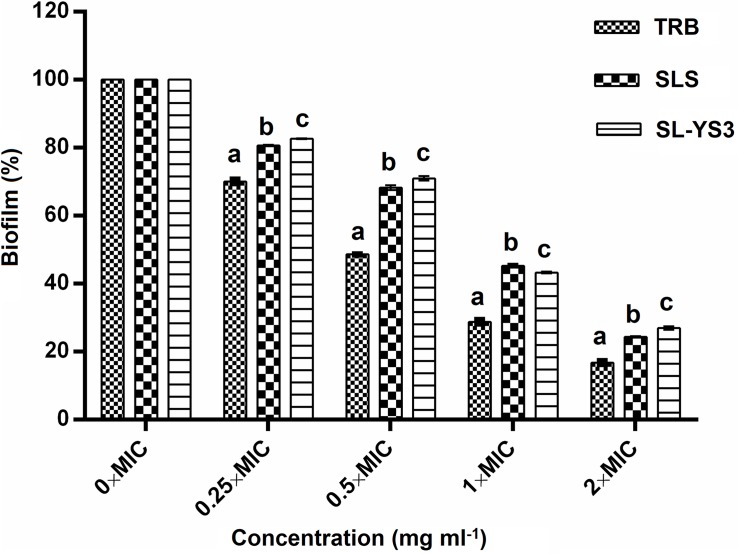
Dispersal of pre-formed biofilms of *T. mentagrophytes* by the sophorolipid produced by *R. babjevae* YS3 (SL-YS3), sophorolipid standard 1, 4′′-sophorolactone 6′,6′′-diacetate (SL-S), and terbinafine (TRB). Data are percentage of the mean of triplicates with respect to control. Error bars represent standard deviation. Different letters within each concentration indicate significantly different values as per ANOVA-LSD.

The effect of a range of concentrations of SL-YS3 on the disruption of biofilms formed on polystyrene microtiter plates was evaluated with CV staining (Figure 6). SL-S and TRB were included for comparison purposes. A dose-dependent reduction of the biofilms was observed (*F*_4_,_40_ = 184.621, *P* < 0.000). Although the biofilms were not completely eradicated at 2 × MICs of SL-YS3 (2 mg ml^–1^) similar to TRB (0.062 mg ml^–1^) and SL-S (2 mg ml^–1^), a significant reduction (*P* < 0.000, LSD) was noted.

#### SEM and CLSM

**FIGURE 7:**
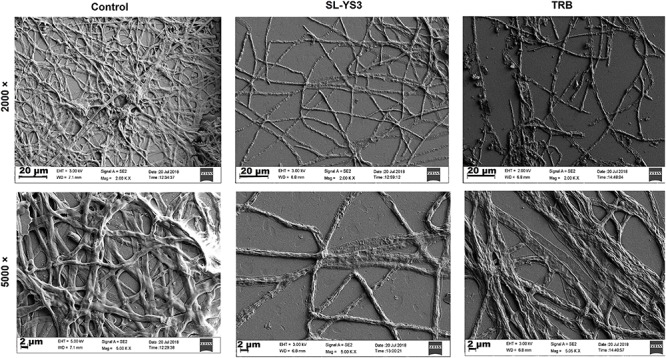
Scanning electron microscopic (SEM) images of the effect of sophorolipid produced by *R. babjevae* YS3 and terbinafine (TRB) on mature biofilms of *T. mentagrophytes*. Scale bar = 20 μm (2000), 2 μm (5000).

**FIGURE 8:**
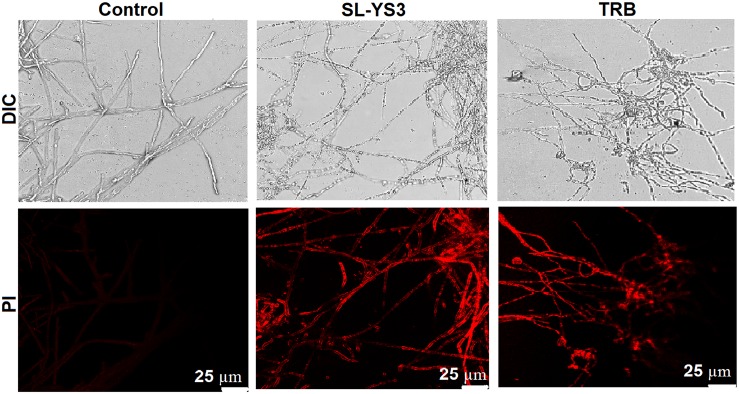
Confocal laser scanning microscopic (CLSM) images of the effect of sophorolipid produced by *R. babjevae* YS3 and terbinafine (TRB) on mature biofilms of *T. mentagrophytes*. Scale bar = 25 μm.

Scanning electron microscopy and CLSM images (Figures 7, 8) showed significant disruption of *T. mentagrophytes* biofilms and distinct structural differences on exposure to SL-YS3 and TRB in comparison to the untreated control. SEM micrographs (Figure 7) of treated samples depicted clear morphological variations like flattened and wrinkled hyphae, substantial reduction of the extracellular matrix, and loosely arranged mycelia. Confocal imaging (Figure 8) revealed increased red fluorescence due to the permeation of PI on treatment with SL-YS3 and TRB, indicating cell death. In contrast, the untreated control samples exhibited no fluorescence indicating healthy cells.

### Assessment of the Therapeutic Efficacy of SL-YS3 in Mice

**FIGURE 9:**
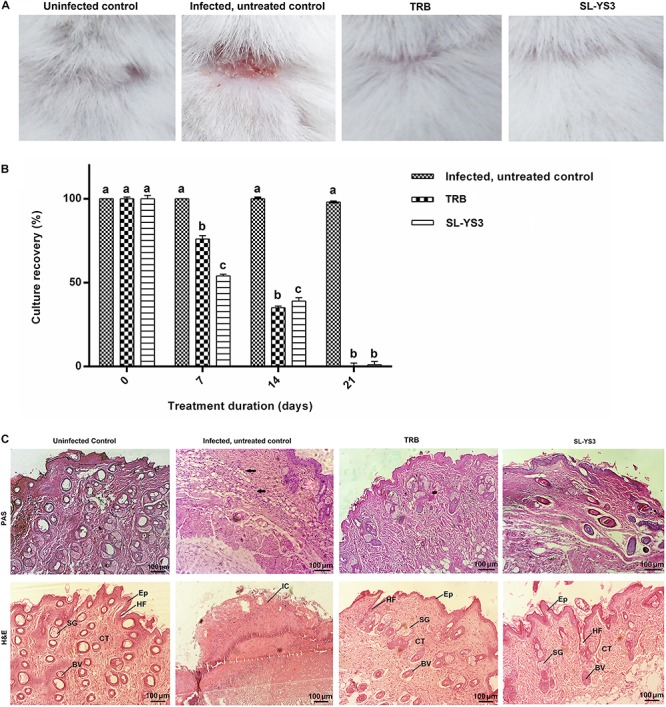
Therapeutic efficacy of sophorolipid produced by *R. babjevae* YS3 (SL-YS3) on experimentally induced dermatophytosis in mice infected with *T. mentagrophytes*. **(A)** Macroscopic observation of the infected sites of the mice treated with 1 mg ml^–1^ (w/v) SL-YS3 and terbinafine (TRB) at the end of 21 days of treatment in comparison to the uninfected and infected, untreated control. **(B)** Culture recovery (%) of *T. mentagrophytes* from skin samples collected at different intervals of treatment period (0, 7, 14, 21 days) and cultured to determine the therapeutic efficacy in eradication of the fungus. Error bars represent standard deviation. Different letters indicate significantly different values as per ANOVA-LSD. **(C)** Histopathology of skin samples collected from sophorolipid (SL-YS3) and terbinafine (TRB) treated groups, stained with hematoxylin & eosin (H&E) and Periodic acid- Schiff (PAS) stains. SG, sweat glands; Ep, epidermis; HF, hair follicle; BV, blood vessel; CT, collagenase tissue; IC, inflammatory cells. Magnification = 10 ×, Scale bar = 100 μm.

Figure 9A depicts the appearance of mice skin after 21 days to macroscopically determine the clinical efficiency of SL-YS3. The effect was comparable to TRB in eradicating the fungal infection, as evident from the increased healing of skin lesions and the restoration of hair growth relative to the infected, untreated control group.

#### Fungal Culture Recovery

When culturing skin scrapings from the animals of different groups, it was found that SL-YS3 and TRB were effective in a time-dependent manner against the pathogen (*F*_3_,_28_ = 399.708, *P* < 0.000) with comparable efficiencies (*F*_15_,_16_ = 1.231, *P* = 0.342). The complete eradication of the fungus was evident from the absence of culture-positive skin tissue collected at the end of the 21-day treatment period (Figure 9B). In contrast, the control model exhibited 100% culture recovery from the skin scrapings collected throughout the experimental period of 21 days.

#### Histopathology

Skin tissue of the SL-YS3 and TRB treated animals stained with H&E showed tissue regeneration with better organization of the dermal ultrastructure evidenced by re-epithelialization, the formation of collagenase tissue, the presence of hair follicles and sebaceous glands. Very mild inflammatory cells were observed in these groups (Figure 9C, H&E). Skin tissue from the uninfected animals showed a prominent epidermis with no inflammatory cell infiltrate. All the adnexal cell structures were visible along with neovascularization. The infected, untreated control animals showed complete disruptions of epithelium along with inflammatory cell infiltrate. PAS staining was performed to visualize the presence of fungal elements (Figure 9C, PAS). The PAS-positive reaction was visible in the infected, untreated control samples, which were absent in the TRB and SL-YS3 treated samples indicating the eradication of the fungus by the end of the treatment duration. The results of this study confirmed the antifungal mediated wound healing ability of SL-YS3, similar to that of‘ TRB.

## Discussion

Identification and development of novel antifungal agents is an important goal of current anti-infective research, which could represent structural templates for structure-activity relationship studies, thus providing more information to optimize potential new antifungal agents (Ngo et al., 2016). SLs are known antimicrobial agents; however, the majority of the reports describe only their antibacterial effect and tissue healing properties (De Rienzo et al., 2016; Lydon et al., 2017). The inhibitory effect of SLs against human pathogenic filamentous fungi is largely unexplored. In our current study, we have investigated the antifungal activity of SL against *T. mentagrophytes* in experimentally induced dermatophytosis in a mouse model. To the best of our knowledge, this is the first report describing the antidermatophytic activity of SLs.

Although another species of *Rhodotorula*, i.e., *R. bogoriensis* has been well-documented as a SL producer (Nuñez et al., 2004; Ribeiro et al., 2012), *R. babjevae* has been described as a biosurfactant producer for the very first time in our previous report (Sen et al., 2017). However, a couple of studies involving *R. babjevae* strains UCDFST 04-877 (Cajka et al., 2016) and Y-SL7 (Guerfali et al., 2019) described the biosurfactant as polyol esters of fatty acids (PEFA). Both SLs and PEFA are known to have a very similar molecular formulae but with different chemical structures. Although the PEFA were described to contain a similar fatty acid component as those observed in the case of *R. babjevae* YS3, the main difference was the glycan part that comprised of mannitol and arabitol instead of the sophorose found in SLs. As *R. babjevae* is largely under characterized, there is a genuine possibility that strains belonging to different geographic locations, when cultured under different sets of conditions, might produce different types of biosurfactants. Consequently, to validate our previous findings, SL-YS3 was characterized further using GC–MS and UPLC-ESI-MS to determine the composition of the hydrophobic (fatty acid component) and hydrophilic moiety (sugar component), respectively. In GC–MS analyses of the FAMEs, it was revealed that SL-YS3 comprised of congeners containing six different fatty acids. The most abundant fatty acids in SL-YS3 were palmitic acid (C16:0), stearic acid (C18:0), and lauric acid (C11:0). The stearic acid side chain was also observed in the SL-S analyzed under the same set of conditions and exhibited a similar retention time and mass spectrum. The UPLC-ESI-MS analyses of the sugar components of both the samples confirmed glucose as the sole monomer present, and no other sugars were detected, confirming SL-YS3 to be an SL type of biosurfactant. The mass spectral analyses substantiated the same upon comparison with previous literature (Macášek et al., 2003; Pathak et al., 2015).

SL-YS3 exhibited disruptive activity on mycelial proliferation and conidial germination of *T. mentagrophytes*. This is of importance as in dermatophytosis, conidia attach to the epithelial cells for germination and initiate an invasion of the stratum corneum layer. Successful hyphal penetration into deeper layers of the epidermis is essential for the establishment and maintenance of infection as the outer tissue layers are lost at regular intervals (de Oliveira Pereira et al., 2013). The activity of biosurfactants is dependent on their structures, which, in turn, promote variations in their action potential against the target organisms (Das et al., 2014). Among other factors, antimicrobial activities of SLs depend on the types of fatty acid moieties (Zhang et al., 2014). Lactonic forms of SLs show superior biological activity as compared to their acidic forms (Borsanyiova et al., 2016). It was observed that the MIC values were the same for both SL-YS3 and SL-S. SL-YS3 comprises predominantly lactonic congeners, and so does SL-S, which is exclusively lactonic in nature. Both these SLs also contain di-acetylated SL-C_18_ as a common component, which might account for the similar MIC value of both SL-YS3 and SL-S (lactonic SL) as reportedly, the presence of acetyl groups enhances the biological activity of SLs (Shah et al., 2005b).

The fungal cell wall is a dynamic structure that protects fungal protoplasts from external osmotic shocks and defines fungal morphogenesis. Changes in the organization or functional disruption of the fungal cell wall induced by antifungal agents are involved in fungal death (de Oliveira Pereira et al., 2013). Many of the biosurfactant groups can lyse the fungal membranes owing to their ability to bind to cholesterol, with which they share common structural features (Otzen, 2017). To determine if SL-YS3 affects the cell membrane of *T. mentagrophytes*, microscopic evaluation of SL-YS3 treated mycelia was performed. TRB was included as the standard drug for this study as it is the first line of drug for the treatment of tinea corporis and tinea cruris due to its favorable mycological and pharmacokinetic profile (Bhatia et al., 2019). TRB acts by inhibiting the enzyme squalene epoxidase, thereby inhibiting ergosterol synthesis. This leads to the accumulation of a large amount of squalene, which leads to increased membrane permeability and cell death (Sahni et al., 2018).

**FIGURE 10:**
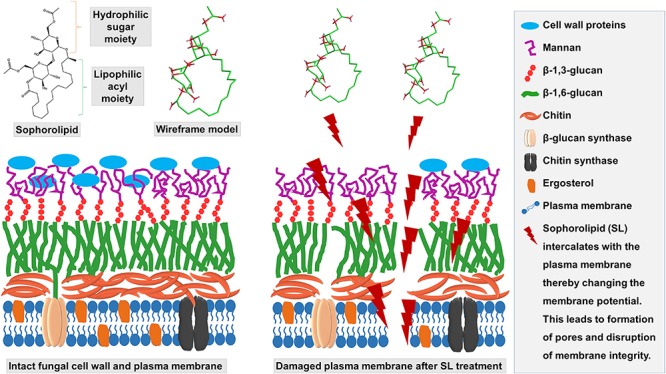
Schematic depicting the mechanism of action of SL-YS3 on mycelial membrane of *T. mentagrophytes*. Owing to the amphiphilic nature, SL-YS3 might alter the permeability of the cell membrane of *T. mentagrophytes*, interfering with the ion transport leading to cell membrane rupture and cell death.

As expected, the morphological and topographic changes visualized with SEM and AFM, depicted the ultrastructural damage of the treated mycelia of *T. mentagrophytes* as compared to the untreated samples. Previously, Sun et al. (2004) demonstrated the membrane permeabilization effect of an SL produced by *Starmerella bombicola* against algal cells, to mitigate the deleterious effect of algal blooms. The membrane integrity and viability can be determined by cellular ability to exclude or penetrate dyes by live cells or damaged and necrotic cells (Coder, 2001). PI, a membrane-impermeable fluorescent dye that binds to nucleic acids, is widely used to differentiate cells that have damaged plasma membranes from healthy ones. A PI uptake assay, to differentiate between live and dead cells as visualized by CLSM, confirmed the damage to the membrane structure when treated with SL-YS3. These observations support the hypothesis that SL can alter the membrane permeability, causing irreversible damage to its integrity, and is lucid evidence for antifungal activity of SL-YS3 (Shah et al., 2005a). It has been found that antifungal compounds with a lipophilic character increase the fluidity and permeability of the cell membrane of microorganisms, interfering with ion transport and subsequently unbalances ion transport (Di Pasqua et al., 2007). This, in turn, leads to the inhibition of microbial growth, cell lysis, or cell death. SL-YS3 has a majority of hydrophobic lactonic congeners (five homologs) as compared to hydrophilic acidic congeners (two homologs) giving it more of a lipophilic property (Sen et al., 2017)_ENREF_40. Therefore, SL-YS3 might exert its effect by altering the permeability of the target fungal cell membrane causing cell death (Figure 10).

Biofilms are the preferred mode of growth in the majority of fungal infections and are difficult to treat due to their resistance to antimicrobials (Quinn et al., 2013). Therefore, the treatment of infections involving biofilms becomes challenging and attracts significant attention. SL-YS3 was effective in dispersing biofilms formed by *T. mentagrophytes* at 2 × MIC concentration. It has been reported that the antifungal drug concentrations required to reduce metabolic activity were 30 to 2,000 times higher than the corresponding MICs (Ali et al., 2016). Haque et al. (2016) reported that an SL produced by *Starmerella bombicola* MTCC 1910 showed 80% biofilm eradication at a concentration which was fourfold higher to the MIC_80_, i.e., the concentration at which 80% inhibition of planktonic cells was observed. SEM and CLSM studies of the biofilms formed on coverslips showed that the biofilms were sensitive to SL-YS3, as evident from the reduction of the biomass and ECM, loose hyphal arrangement (SEM), and increased fluorescence in the treated samples (CLSM). Compounds that can remove the ECM may be an effective strategy to disperse biofilms, disaggregate the cells, and disrupt the pathogenic environment. This might be due to the surfactant action of SL-YS3. Recalcitrance and resistance have been frequently observed in dermatophytic infections, especially onychomycosis frequently caused by *T. mentagrophytes* that have been related to the formation of biofilms (Burkharta et al., 2002). The biofilm eradication activity exhibited by SL-YS3 is interesting as the ability of any antifungal to penetrate existing biofilms is an important trait for any antimicrobial as it affects drug efficacy and the potential for the emergence of antimicrobial resistance.

Preclinical studies with SL-YS3 as the antifungal agent-induced complete recovery post-treatment in a mouse model of dermatophytosis caused by *T. mentagrophytes*, advocating the antifungal potential of SL-YS3. To evaluate *in vivo* activity, experimental infection with *T. mentagrophytes* in mouse models to mimic dermatophytosis has been successfully used for evaluation of the activities of several antifungal agents (Hay et al., 1983; Almeida et al., 2017). The culture of the tissue samples yielded a negative result in SL-YS3, and TRB treated groups, which could be considered clinically cured in the therapeutic process.

The histopathological examination with PAS clearly showed the presence of fungal elements in the untreated skin tissue. The treated sections showed similar therapeutic effects of SL-YS3 and TRB on the regeneration of the impaired epidermis of the infected mice. SL-YS3 also showed its ability to regulate collagen deposition together with proper matrix and spatial arrangement, thereby contributing to the healing of the infected skin tissue as compared to the untreated control. This is consistent with Lydon et al. (2017), who concluded that the application of purified SLs is compatible with healing wounds and could be beneficial in preventing wound contamination or infection with opportunistic bacterial pathogens.

However, the results obtained in this study might vary in cases of onychomycosis caused by *T. mentagrophytes*. Topical application in such cases is often not satisfactory, particularly due to poor penetration of antifungals through the nail plate. The physicochemical properties of the drug and the characteristics of the formulations, such as molecular weight, lipophilicity, affinity to keratin etc., influence the permeation (Angelo et al., 2017). As such, evaluation of the biological activity of SL-YS3 *in vitro* and experimental models for onychomycosis would be necessary.

## Conclusion

SL-YS3 could effectively cure dermatophytosis caused by *T. mentagrophytes*. The compound interacts with the cell membrane of the pathogen and exerts its effect by disturbing the membrane integrity. SL-YS3 was also effective against the biofilms formed by *T. mentagrophytes* and also showed therapeutic efficiency in studies based on mouse models. The results are suggestive of the prospect of SL-YS3 as an effective and non-toxic alternative treatment for dermatophyte infections to substitute the synthetic antifungals currently in use. Nevertheless, as only a single strain has been used in our study, the results should form the basis for further investigations encompassing several clinical isolates and resistant strains.

## Data Availability Statement

The datasets generated for this study are available on request to the corresponding author.

## Ethics Statement

The animal study was reviewed and approved by the Institutional Animal Ethics Committee (IAEC) of IASST, Guwahati.

## Author Contributions

SS and SD designed the entire study. SS carried out all the experiments and wrote the manuscript. SB helped with the experiments, analyzed the statistical data, and contributed to the manuscript writing. RK helped in the design and execution of the animal model. AB provided valuable input during the experimental design. All the authors have reviewed the manuscript.

## Conflict of Interest

The authors declare that the research was conducted in the absence of any commercial or financial relationships that could be construed as a potential conflict of interest.
